# Volume and Intensity of Walking and Risk of Chronic Low Back Pain

**DOI:** 10.1001/jamanetworkopen.2025.15592

**Published:** 2025-06-13

**Authors:** Rayane Haddadj, Anne Lovise Nordstoga, Tom Ivar Lund Nilsen, Eivind Schjelderup Skarpsno, Atle Kongsvold, Mats Flaaten, Jasper Schipperijn, Kerstin Bach, Paul Jarle Mork

**Affiliations:** 1 Department of Public Health and Nursing, Norwegian University of Science and Technology, Trondheim, Norway; 2 Department of Neuromedicine and Movement Science, Norwegian University of Science and Technology, Trondheim, Norway; 3 Clinic of Emergency Medicine and Prehospital Care, St. Olav’s Hospital, Trondheim, University Hospital, Trondheim, Norway; 4 Department of Sports Science and Clinical Biomechanics, University of Southern Denmark, Odense, Denmark; 5 Department of Computer Science, Norwegian University of Science and Technology, Trondheim, Norway

## Abstract

**Importance:**

Chronic low back pain (LBP) is a prevalent and costly condition, and regular physical activity may reduce its risk. Walking is a common and accessible form of physical activity, but its association with the risk of chronic LBP is unclear.

**Objective:**

To examine whether accelerometer-derived daily walking volume and walking intensity are associated with the risk of chronic LBP.

**Design, Setting, and Participants:**

This prospective population-based cohort study used data from the Trøndelag Health (HUNT) Study in Norway, with a baseline in 2017 to 2019 and follow-up in 2021 to 2023. The study included individuals without chronic LBP at baseline and with at least 1 valid day of device-measured walking.

**Exposure:**

Daily walking volume (minutes per day) and walking intensity, expressed as metabolic equivalent of task (MET) per minute.

**Main Outcomes and Measures:**

The primary outcome was self-reported chronic LBP at follow-up, defined as pain lasting 3 months or longer in the past 12 months. Poisson regression was used to estimate adjusted risk ratios (RRs) with 95% CIs of chronic LBP according to daily walking volume and mean walking intensity.

**Results:**

A total of 11 194 participants aged 20 years or older (mean [SD] age, 55.3 [15.1] years; 6564 women [58.6%]) were included in the analysis. At follow-up (mean [SD] follow-up time, 4.2 [0.3] years), 1659 participants (14.8%) reported chronic LBP. Continuous measures of both walking volume and walking intensity were inversely associated with the risk of chronic LBP using restricted cubic splines models. Compared with participants walking less than 78 minutes per day, those walking 78 to 100 minutes per day had an RR for chronic LBP of 0.87 (95% CI, 0.77-0.98), those walking 101 to 124 minutes per day had an RR of 0.77 (95% CI, 0.68-0.87), and those walking 125 minutes or more per day had an RR of 0.76 (95% CI, 0.67-0.87). Compared with a mean walking intensity of less than 3.00 MET per minute, participants with walking intensity of 3.00 to 3.11 MET per minute had an RR for chronic LBP of 0.85 (95% CI, 0.75-0.96), those with walking intensity of 3.12 to 3.26 MET per minute had an RR of 0.82 (95% CI, 0.72-0.93), and those with walking intensity greater than or equal to 3.27 MET per minute had an RR of 0.82 (95% CI, 0.72-0.93). After mutual adjustment, the association remained largely similar for walking volume but was attenuated for walking intensity.

**Conclusions and Relevance:**

In this cohort study, daily walking volume and walking intensity were inversely associated with the risk of chronic LBP. The findings suggest that walking volume may have a more pronounced benefit than walking intensity.

## Key Points

**Questions:**

Are daily walking volume and walking intensity associated with the risk of chronic low back pain?

**Findings:**

This population-based cohort study including 11 194 participants found an inverse and nonlinear association between walking volume and the risk of chronic low back pain. Walking for more than 100 minutes per day was associated with a 23% lower risk of chronic low back pain compared with walking less than 78 minutes per day; walking intensity was also associated with risk of chronic low back pain but to a lesser degree than walking volume.

**Meaning:**

These findings indicate that policies and public health strategies promoting walking may help to reduce the burden of chronic low back pain.

## Teaser

This cohort study examines whether daily walking volume and walking intensity are associated with the risk of chronic lower back pain.

## Introduction

Low back pain (LBP) affects people of all ages and is the leading cause of functional health loss, estimated to account for 7.7% of all years lived with disability.^1,2^ In the US, back pain is the most common type of chronic pain, and LBP accounts for the highest health care spending along with neck pain.^3,4^ The burden of LBP is projected to increase in the coming decades, posing a substantial challenge to the sustainability of health care systems.^1,5^ To reduce this burden, it is essential to identify modifiable factors that can be targeted through policy and preventive actions.

Guidelines for managing chronic LBP recommend remaining physically active.^6,7^ However, there are no explicit recommendations regarding physical activity for the primary prevention of chronic LBP.^8,9^ Walking, the most common form of leisure-time physical activity among adults, is associated with a lower risk of various noncommunicable diseases and conditions.^10,11^ However, the association between walking and the risk of chronic LBP remains largely unexplored.

A recent randomized clinical trial^12^ demonstrated that a walking and educational program reduced LBP recurrence in individuals who had recently recovered from an episode of nonspecific LBP. Moreover, systematic reviews and meta-analyses^13,14,15^ of randomized clinical trials report evidence supporting a primary preventive effect of exercise (eg, strength, endurance, and stretching) on LBP among asymptomatic individuals. Notably, none of the studies included in these reviews evaluated the effect of walking. In addition, prospective cohort studies^16,17,18^ have provided mixed results regarding the preventive effect of physical activity. Thus, it remains unclear whether walking reduces the risk of chronic LBP. The aim of this study was, therefore, to examine whether daily walking volume and walking intensity are associated with the risk of chronic LBP.

## Methods

### Study Population

**Figure 1.**
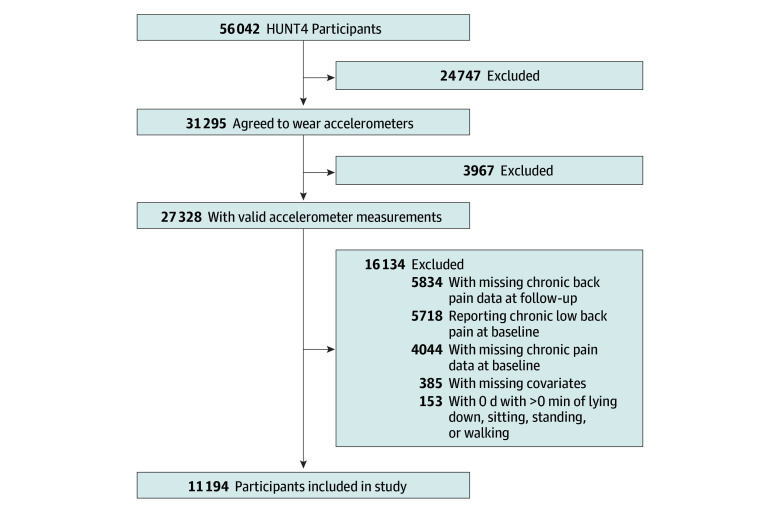
Flowchart Diagram of Inclusion in the Study

This cohort study used data from the population-based Trøndelag Health (HUNT) Study, Norway.^19^ Baseline data were obtained from HUNT4 (2017-2019), in which all inhabitants in the region of Nord-Trøndelag aged 20 years or older were invited to participate. A total of 56 042 of 103 800 individuals (54.0%) agreed to participate, met for a clinical examination, and answered questionnaires on lifestyle and health. All participants in HUNT4 were invited to the HUNT COVID (2021-2023) follow-up survey, of whom 32 743 individuals (58.4%) accepted the invitation. Participants with missing pain data, invalid accelerometer data, prevalent chronic LBP at baseline, or missing data on covariates were excluded from the analyses (Figure 1). This study was approved by the Regional Committee for Ethics in Medical Research, Mid-Norway, and all participants provided written informed consent. This study is reported in accordance with the Strengthening the Reporting of Observational Studies in Epidemiology (STROBE) reporting guideline.^20^

### Walking Volume and Walking Intensity

In HUNT4, 31 295 participants agreed to wear 2 tri-axial AX3 accelerometers (Axivity, Ltd) for 7 days. The procedure for data collection has been described in detail elsewhere.^21^ Briefly, 1 accelerometer was placed centrally on the right thigh approximately 10 cm above the upper border of the patella, and 1 was positioned on the third lumbar segment on the lower back. Walking along with other key activity types and postures were classified over 5-second epochs using an eXtreme Gradient Boosting (XGBoost) machine learning classifier.^22,23^ All 5-second epochs with walking were further classified into slow (≤4 km/hour), moderate (4.1-5.4 km/hour), and brisk (5.5-6.4 km/hour) walking, using a machine learning classifier validated against instantaneous walking speed.^24^ We excluded days with zero minutes of either lying down, sitting, standing, or walking and included all participants with 1 or more days with valid accelerometer data (ie, midnight-to-midnight recording), irrespective of weekdays or weekend days. Daily walking volume (ie, minutes per day) was computed by averaging the total volume of walking by number of days with valid data. Walking intensity was derived as the mean metabolic equivalent of task (MET) per minute according to estimated MET values for the slow, moderate, and brisk walking, respectively (ie, 2.8, 3.8, and 4.8 MET per minute) (eTable 1 in Supplement 1).^25^

### Chronic LBP

Chronic LBP was assessed by a modified version of the Standardized Nordic Questionnaire.^26^ In HUNT4 and HUNT COVID, participants were asked, “During the last year, have you had pain and/or stiffness in your muscles or joints that lasted for at least 3 consecutive months?” with yes or no response options. If they answered yes, participants were asked, “Where have you had this pain or stiffness?” Participants were considered to have chronic LBP if they answered yes to the first question and reported pain in the low back to the second question.

### Other Variables

Possible confounders were identified from a directed acyclic graph and included variables that are associated with both the exposure and outcome (eFigure 1 in Supplement 1). All associations were adjusted for age (years), sex (male or female), education (secondary school, 1-2 years of academic or vocational school, 3 years of academic or vocational school, 3-4 years of vocational school or apprentice, university <4 years, and university ≥4 years), annual household income in US dollars (<$25 000, $25 000-$45 000, $45 100-$75 000, $75 100-$100 000, and ≥$100 000), employment status (employed or nonemployed), smoking status (never, former, or current), and depression (no or yes, defined as a cutoff score of ≥7 on the depression subscale of the Hospital Anxiety and Depression Scale).^27^

### Statistical Analysis

Poisson regression was used to estimate adjusted risk ratios (RRs) of chronic LBP associated with quarters of walking volume and walking intensity. Restricted cubic splines with 3 knots placed at the 10th, 50th, and 90th percentile of the distribution were used to assess the dose-response association of daily walking volume and mean walking intensity with the risk of chronic LBP.^28^ Nonlinearity of the dose-response association was assessed in 2 steps. First, a Wald test including all terms related to the exposure (ie, walking volume or intensity and all cubic terms defined by the knots) was conducted. Second, if this test was statistically significant (ie, 2-sided *P* < .05), a second Wald test was performed only including the nonlinear term.^29^ We estimated the joint association of daily walking volume and walking intensity with the risk of chronic LBP using the first quarter of walking volume and walking intensity as reference. We also conducted an analysis of the association between mean walking intensity and risk of chronic LBP stratified by quarters of daily walking volume. In an additional analysis, we assessed for possible effect modification by age and sex in a likelihood ratio test of a product term between walking volume and either age (<65 vs ≥65 years) or sex (female or male), as well as in stratified analyses. The cutoff for age of 65 years was chosen since this is the typical age of retirement in Norway and could, thus, capture work-related differences in walking volume. To try to distinguish the independent contributions of volume and intensity of walking to the risk of chronic LBP, we conducted supplementary analyses where these variables were mutually adjusted. The precision of the RRs was assessed by 95% CIs using robust variance estimation.

Sensitivity analyses were performed to address potential biases and verify the robustness of our findings. To assess possible reverse causation, we conducted 4 separate analyses where we restricted the study sample by excluding participants who at baseline reported (1) any chronic pain (ie, responded yes to the question, “During the last year, have you had pain and/or stiffness in your muscles or joints that lasted for at least 3 consecutive months?”), (2) moderate to very strong pain intensity on a 6-level scale (ranging from no pain to very strong pain) during the last 4 weeks, (3) poor or not so good health status on a 4-level scale (ranging from poor health to very good health), and (4) a history of diabetes, cancer, or cardiovascular disease. To assess the potential measurement error associated with short accelerometer recording period, we performed an analysis including only participants with 4 days or more of valid accelerometer data, irrespective of weekdays or weekend days. Since body mass index (BMI; calculated as weight in kilograms divided by height in meters squared) could both confound and mediate the effect of walking on the risk of chronic LBP due to the likely bidirectional effect, we controlled for BMI in an additional sensitivity analysis. To consider potential unmeasured confounding associated with other physical activity types, we conducted an analysis further adjusting for the summed daily volume of running and cycling. To assess the association between walking and the risk of more severe chronic LBP, we conducted an analysis restricting the outcome definition to chronic LBP among those reporting pain-related limitations in leisure-time activities. Finally, we computed E-values for each RR and its 95% CI limit closest to the null. The E-value indicates the minimum magnitude of association that an unmeasured confounder must have with both the exposure and the outcome, conditional on measured covariates, to fully explain the observed association between exposure and outcome.^30^

All analyses were conducted using Stata statistical software version 18.5 (StataCorp). All relevant Stata code and documentation can be accessed via the project depository.^31^

## Results

Table 1 presents the baseline characteristics of the 11 194 participants included in the analysis, stratified by quarters of daily walking volume. The mean (SD) age of participants was 55.3 (15.1) years, with 6564 (58.6%) being women. The accelerometers were worn for a mean (SD) of 5.7 (1.0) days at the baseline survey, and the mean (SD) walking volume was 103.8 (36.2) minutes per day. After a mean (SD) follow-up period of 4.2 (0.3) years, 1659 participants (14.8%) reported chronic LBP.

**Table 1.** Baseline Characteristics of the Study Population Stratified by Quarters of Daily Walking Volume

Characteristic	Participants, No. (%)				
	Total (N = 11 194)	Q1 daily walking volume <78 min/d (n = 2800)	Q2 daily walking volume 78-100 min/d (n = 2800)	Q3 daily walking volume 101-124 min/d (n = 2796)	Q4 daily walking volume ≥125 min/d (n = 2798)
Age, mean (SD), y	55.3 (15.1)	57.4 (17.3)	54.6 (15.3)	54.7 (14.3)	54.6 (13.1)
Sex					
Female	6564 (58.6)	1570 (56.1)	1659 (59.2)	1703 (60.9)	1632 (58.3)
Male	4630 (41.4)	1230 (43.9)	1141 (40.8)	1093 (39.1)	1166 (41.7)
Days of accelerometry, mean (SD)	5.7 (1.0)	5.6 (1.1)	5.7 (1.0)	5.7 (0.9)	5.7 (1.1)
Walking intensity, mean (SD), MET per minute	3.1 (0.2)	3.1 (0.2)	3.1 (0.2)	3.2 (0.2)	3.2 (0.2)
Education					
Primary school	719 (6.4)	275 (9.8)	170 (6.1)	147 (5.3)	127 (4.5)
Secondary school	5029 (44.9)	1287 (46.0)	1211 (43.2)	1190 (42.6)	1341 (47.9)
University	5446 (48.7)	1238 (44.2)	1419 (50.7)	1459 (52.2)	1330 (47.5)
Yearly household income, $US					
<25 000	475 (4.2)	219 (7.8)	115 (4.1)	76 (2.7)	65 (2.3)
25 000-45 000	1969 (17.6)	661 (23.6)	459 (16.4)	431 (15.4)	418 (14.9)
45 100-75 000	3418 (30.5)	865 (30.9)	868 (31.0)	822 (29.4)	863 (30.8)
75 100-100 000	2881 (25.7)	571 (20.4)	719 (25.7)	792 (28.3)	799 (28.6)
>100 000	2451 (21.9)	484 (17.3)	639 (22.8)	675 (24.1)	653 (23.3)
Employment status					
Employed	7512 (67.1)	1471 (52.5)	1933 (69.0)	1989 (71.1)	2119 (75.7)
Nonemployed	3682 (32.9)	1329 (47.5)	867 (31.0)	807 (28.9)	679 (24.3)
Smoking status					
Never smoker	5284 (47.2)	1213 (43.3)	1315 (47.0)	1362 (48.7)	1394 (49.8)
Former smoker	5165 (46.1)	1340 (47.9)	1290 (46.1)	1278 (45.7)	1257 (44.9)
Current smoker	745 (6.7)	247 (8.8)	195 (7.0)	156 (5.6)	147 (5.3)
Depression^a^					
No	10 090 (90.1)	2433 (86.9)	2539 (90.7)	2547 (91.1)	2571 (91.9)
Yes	1104 (9.9)	367 (13.1)	261 (9.3)	249 (8.9)	227 (8.1)

Abbreviations: Q, quarter; MET, metabolic equivalent of task.

^a^ Depression was assessed using the depression subscale of the Hospital Anxiety and Depression Scale.

### Volume and Intensity of Walking and Risk of Chronic LBP

Table 2 shows the risk of chronic LBP associated with quarters of daily walking volume and average walking intensity. Compared with the reference group, which walked less than 78 minutes per day (first quarter), a reduced risk was observed for those who walked 78 to 100 minutes per day (second quarter, RR, 0.87; 95% CI, 0.77-0.98), 101 to 124 minutes per day (third quarter, RR, 0.77; 95% CI, 0.68-0.87), and 125 minutes or more per day (fourth quarter, RR, 0.76; 95% CI, 0.67-0.87). There was some evidence that the association between walking volume and risk of chronic LBP was greater and more consistent in those aged 65 years and older than in those younger than 65 years (*P* for interaction = .01) (eTable 2 in Supplement 1). There was no evidence of interaction between sex and walking volume (results not shown). In addition, compared with the lowest quarter of average walking intensity (<3.00 MET per minute), a reduced risk was observed for participants with an average walking intensity of 3.00 to 3.11 MET per minute (second quarter, RR, 0.85; 95% CI, 0.75-0.96), 3.12 to 3.26 MET per minute (third quarter, RR, 0.82; 95% CI, 0.72-0.93), and greater than or equal to 3.27 MET per minute (fourth quarter, RR, 0.82; 95% CI, 0.72-0.93). There was no evidence of interaction between age and sex with quarters of mean walking intensity (results not shown).

**Table 2.** Risk of Chronic Low Back Pain at Follow-Up in 2021 to 2023 Associated With Quarters of Daily Walking Volume and Quarters of Mean Walking Intensity at Baseline in 2017 to 2019

Variable	No. of participants/No. of cases	Age-adjusted RR (95% CI)^a^	Multiadjusted RR (95% CI)^b^
Walking volume, min/d			
Q1, <78	2800/519	1.00 [Reference]	1.00 [Reference]
Q2, 78-100	2800/418	0.83 (0.74-0.93)	0.87 (0.77-0.98)
Q3, 101-124	2796/364	0.72 (0.64-0.82)	0.77 (0.68-0.87)
Q4, ≥125	2798/358	0.71 (0.63-0.81)	0.76 (0.67-0.87)
Mean walking intensity, MET per minute			
Q1, <3.00	2799/544	1.00 [Reference]	1.00 [Reference]
Q2, 3.00-3.11	2798/395	0.77 (0.68-0.87)	0.85 (0.75-0.96)
Q3, 3.12-3.26	2799/365	0.72 (0.63-0.82)	0.82 (0.72-0.93)
Q4, ≥3.27	2798/355	0.70 (0.62-0.80)	0.82 (0.72-0.93)

Abbreviations: MET, metabolic equivalent of task; RR, risk ratio; Q, quarter.

^a^ Adjusted for age (continuous).

^b^ Adjusted for age (continuous), sex (female or male), education (primary school, 1-2 years of academic or vocational school, 3 years of academic or vocational school, 3-4 years vocational school or apprentice, university <4 years, and university ≥4 years), annual household income in US dollars (<$25 000, $25 000-$45 000, $45 100-$75 000, $75 100-$100 000, and >$100 000), employment status (nonemployed or employed), smoking status (never, former, or current) and depression (no or yes).

### Joint Association of Volume and Intensity of Walking and Risk of Chronic LBP

Table 3 shows the joint association between daily walking volume and mean walking intensity with the risk of chronic LBP. Within the 3 first quarters of walking volume, the risk of chronic LBP was reduced when the mean walking intensity was above 3.00 MET per minute (ie, second quarter of walking intensity and beyond). This association was less clear among participants within the highest quarter of walking volume (ie, ≥125 minutes per day, fourth quarter). The risk of chronic LBP associated with quarters of mean walking intensity was similar within the 4 different quarters of daily walking volume (eTable 3 in Supplement 1).

**Table 3.** Joint Association of Quarters of Daily Walking Volume and Quarters of Mean Walking Intensity at Baseline in 2017 to 2019 With the Risk of Chronic Low Back Pain at Follow-Up in 2021 to 2023

Daily walking volume and mean walking intensity	No. of participants/No. of cases	Age-adjusted RR (95% CI)^a^	Multiadjusted RR (95% CI)^b^
Q1, walking <78 min/d			
Q1, walking intensity <3.00 MET per minute	1235/282	1.00 [Reference]	1.00 [Reference]
Q2, walking intensity 3.00-3.11 MET per minute	667/110	0.78 (0.64-0.95)	0.86 (0.70-1.05)
Q3, walking intensity 3.12-3.26 MET per minute	536/73	0.65 (0.51-0.82)	0.75 (0.59-0.96)
Q4, walking intensity ≥3.27 MET per minute	362/54	0.73 (0.56-0.96)	0.87 (0.66-1.14)
Q2, walking 78-100 min/d			
Q1, walking intensity <3.00 MET per minute	688/128	0.84 (0.69-1.01)	0.87 (0.72-1.04)
Q2, walking intensity 3.00-3.11 MET per minute	767/112	0.69 (0.56-0.85)	0.77 (0.63-0.94)
Q3, walking intensity 3.12-3.26 MET per minute	702/96	0.65 (0.53-0.81)	0.76 (0.61-0.94)
Q4, walking intensity ≥3.27 MET per minute	643/82	0.61 (0.48-0.77)	0.75 (0.59-0.94)
Q3, walking 101-124 min/d			
Q1, walking intensity <3.00 MET per minute	521/91	0.79 (0.64-0.98)	0.82 (0.66-1.02)
Q2, walking intensity 3.00-3.11 MET per minute	734/92	0.59 (0.47-0.73)	0.67 (0.54-0.84)
Q3, walking intensity 3.12-3.26 MET per minute	740/88	0.56 (0.45-0.71)	0.65 (0.52-0.81)
Q4, walking intensity ≥3.27 MET per minute	801/93	0.55 (0.44-0.69)	0.65 (0.52-0.81)
Q4, walking ≥125 min/d			
Q1, walking intensity <3.00 MET per minute	355/43	0.55 (0.41-0.74)	0.60 (0.44-0.80)
Q2, walking intensity 3.00-3.11 MET per minute	630/81	0.61 (0.48-0.76)	0.68 (0.54-0.86)
Q3, walking intensity 3.12-3.26 MET per minute	821/108	0.62 (0.51-0.77)	0.72 (0.59-0.89)
Q4, walking intensity ≥3.27 MET per minute	992/126	0.60 (0.49-0.73)	0.70 (0.57-0.85)

Abbreviations: MET, metabolic equivalent of task; RR, risk ratio; Q, quarter.

^a^ Adjusted for age (continuous).

^b^ Adjusted for age (continuous), sex (female or male), education (secondary school, 1-2 years of academic or vocational school, 3 years of academic or vocational school, 3-4 years vocational school or apprentice, university <4 years, and university ≥4 years), annual household income in US dollars (<$25 000, $25 000-$45 000, $45 100-$75 000, $75 100-$100 000, and >$100 000), employment status (nonemployed or employed), smoking status (never, former, or current) and depression (no or yes).

### Dose-Response Association of Volume and Intensity of Walking and Risk of Chronic LBP

**Figure 2.**
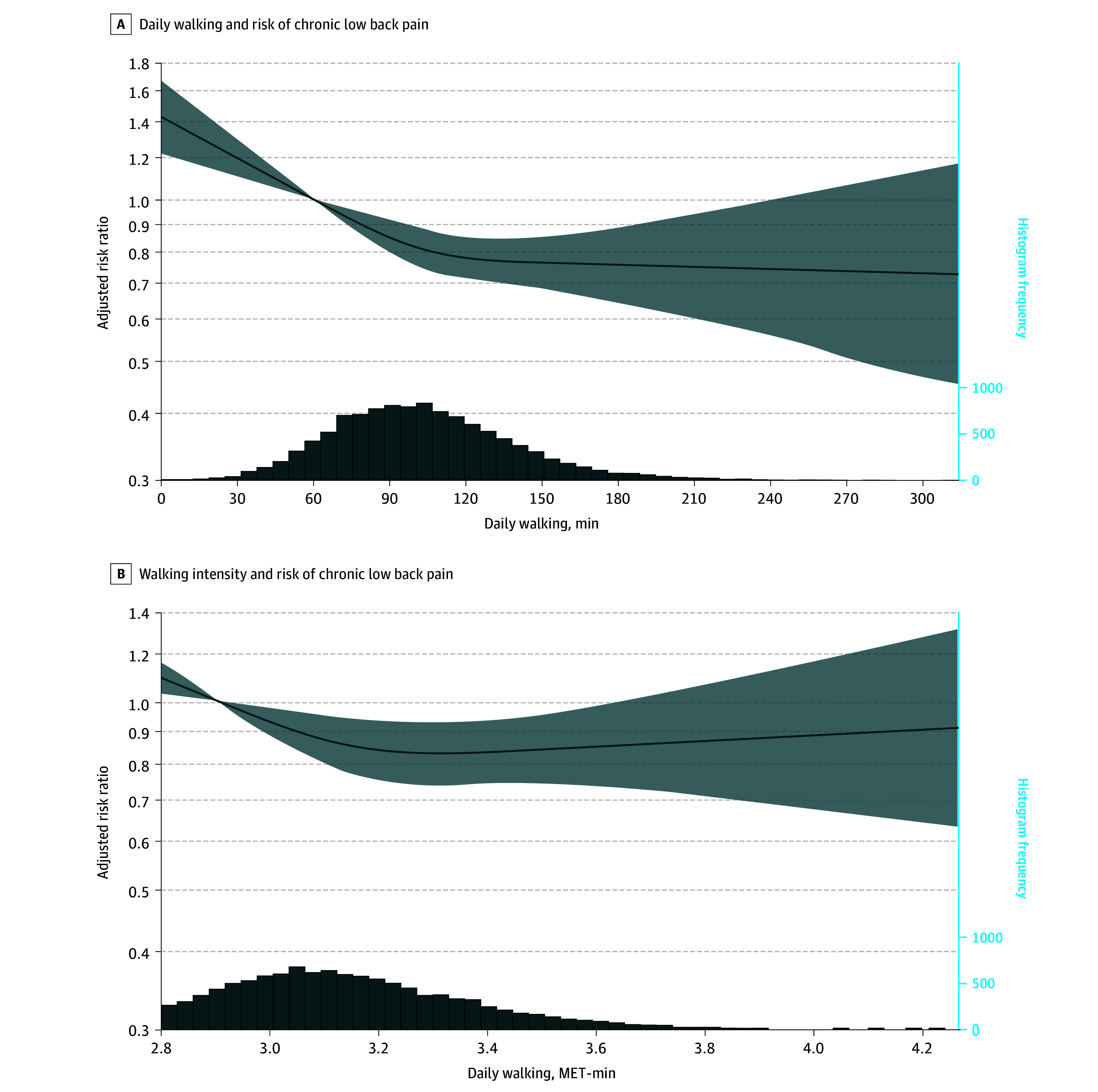
Dose-Response Association of Volume of Daily Walking and Mean Walking Intensity at Baseline in 2017 to 2019 With Risk of Chronic Low Back Pain at Follow-Up in 2021 to 2023 The left y-axis is a log scale with the shaded area representing 95% CIs. Models are adjusted for age, sex, education, income, employment status, smoking status, and depression. Reference is set at the 10th percentile of the distribution. MET indicates metabolic equivalent of task.

Figure 2 illustrates the dose-response associations between the volume and intensity of daily walking and the risk of chronic LBP. The association between daily walking volume and risk of chronic LBP was nonlinear and inverse, showing a steady decline in risk up to approximately 100 minutes per day of walking. A further increase in walking volume was associated with a less pronounced decline in risk. Adjusting for mean walking intensity did not substantially alter this association (eFigure 2A and eTable 4 in Supplement 1). Similarly, a nonlinear association was also observed between mean walking intensity and the risk of chronic LBP. The risk of chronic LBP decreased up to about 3.1 to 3.2 MET per minute, after which there was a slight gradual increase in risk. Adjusting for walking volume attenuated this association (eFigure 2B and eTable 4 in Supplement 1).

### Sensitivity Analyses

Excluding participants with poor or not so good health status at baseline, or participants reporting a history of chronic diseases at baseline, attenuated the association between walking volume and risk of chronic LBP (eTables 5 and 6 in Supplement 1). For walking intensity, excluding participants reporting any chronic pain at baseline, participants reporting moderate or higher pain intensity at baseline, or participants reporting poor or not so good health status at baseline attenuated the association with risk of chronic LBP (eTables 6-8 in Supplement 1). For both walking volume and walking intensity, adjusting for baseline BMI attenuated the association with risk of chronic LBP (eTable 9 in Supplement 1). Other sensitivity analyses did not substantially change the direction or magnitude of the associations between volume or intensity of walking and risk of chronic LBP (eTables 10-12 in Supplement 1). For the independent association between quarters of walking volume and risk of chronic LBP, the E-values ranged from 1.56 (lower 95% CI bound, 1.16) to 1.95 (lower 95% CI bound, 1.58). For the independent association between quarters of mean walking intensity and risk of chronic LBP, the E-values ranged from 1.64 (lower 95% CI bound, 1.25) to 1.75 (lower 95% CI bound, 1.36).

## Discussion

This prospective, population-based cohort study found that daily walking volume was inversely and nonlinearly associated with the risk of chronic LBP. Compared with walking less than 78 minutes per day, those who walked more than 100 minutes per day had a 23% reduced risk of chronic LBP. The reduction in risk of chronic LBP leveled off beyond a walking volume of about 100 minutes per day. A higher mean walking intensity was also associated with a reduced risk of chronic LBP; however, this association was attenuated after adjustment for daily walking volume. To the best of our knowledge, this is the first prospective population-based cohort study to investigate the association between device-measured volume and intensity of walking and the risk of chronic LBP.

Our findings suggest that daily walking volume is more important than mean walking intensity in reducing the risk of chronic LBP. A similar observation was made in a recent meta-analysis^32^ examining the association of total step volume and step intensity (ie, stepping rate) with mortality, which found inconsistent evidence that step intensity is associated with mortality beyond the total step volume. However, it is important to note that we only used 3 cutoffs to classify walking intensity, limiting a more detailed analysis of the association between instantaneous walking intensity and the risk of chronic LBP. Furthermore, participants with higher walking volume tended to exercise more often and reported higher physical work demands (eTable 13 in Supplement 1).

In 2018, *The Lancet* released a 3-part series on LBP, emphasizing the need for research on the prevention of LBP and the identification of modifiable factors that can be implemented in prevention strategies.^33^ Our findings help to bridge this knowledge gap by demonstrating a dose-response association between volume and intensity of walking with the risk of chronic LBP in the general adult population. If confirmed by future research, these results could inform public health strategies aimed at preventing chronic LBP, as well as complementing current guidelines that solely report on physical activity as a secondary prevention tool.^6,34,35,36,37,38,39^ The potential role of walking could be further promoted owing to its ease of implementation, accessibility, and numerous health benefits beyond reducing the risk of chronic LBP.^40,41^

The major strengths of our study include the large population-based sample, the prospective study design, the adjustment for several potential confounders, and the use of device-measured walking, which reduces measurement errors associated with self-reporting. Our results are likely generalizable beyond the Norwegian adult population, as physical inactivity prevalence in Norway is comparable to that observed in other high-income countries.^42^

### Limitations

Our study has several limitations that should be considered when interpreting the results. First, we used a single assessment of daily walking volume and walking intensity, which may have changed over time. A recent study^43^ with 1-year continuous device-based measurement of physical activity indicated that 6 days of measurements are sufficient to achieve a reliable measurement of light physical activity, whereas 10 days are needed for moderate-to-vigorous physical activity. Therefore, considering that the device-measured walking was obtained for a mean of 5.7 days and that most of the accumulated walking volume corresponds to light physical activity, it is unlikely that extending this measurement period would significantly alter our results. Our sensitivity analysis support this, showing that restricting the study sample to participants with 4 or more valid days of measurements did not change the magnitude of the associations between walking volume and intensity with the risk of chronic LBP. Second, the observational study design limits our ability to establish causal inference, and we cannot rule out that volume and intensity of walking at baseline are predictors rather than risk factors for chronic LBP. However, sensitivity analyses excluding participants with other chronic pain, moderate or higher pain intensity, poor or not so good health, or chronic disease history suggest that reverse causation is unlikely to have a strong influence on our results. Nevertheless, we lack information on the progression of chronic LBP and the emergence of new risk factors during the follow-up period. Third, residual confounding may still be present, although the computed E-values suggest a minimal impact on the observed associations. Fourth, the analytical sample tended to be older, more educated, and had higher household income than the total HUNT4 population (eTables 14-16 in Supplement 1). However, whether representativeness is important to obtain unbiased estimates is questionable.^44^ Fifth, the assessment of chronic LBP was based on participants’ recollection of LBP in the past 12 months, and misclassification is possible. However, unless such misclassification was differential between the exposure categories, it would likely underestimate rather than overestimate the association between walking and the risk of chronic LBP.

## Conclusions

In this prospective population-based cohort study, a higher daily walking volume was associated with a lower risk of chronic LBP. Higher walking intensity was associated with less pronounced benefits. These findings suggest that policies and public health strategies promoting walking could help to reduce the occurrence of chronic LBP.

## Notes

Supplement 1. **eTable 1.** Selected conversion values for walking intensity
eReference
**eFigure 1.** Directed acyclic graph for covariate selection **eTable 2.** Risk of chronic low back pain associated with quarters of daily walking volume stratified by age **eTable 3.** Risk of chronic low back pain associated with quarters of mean walking intensity stratified by quarters of daily walking volume **eFigure 2.** Dose-response association of volume of daily walking and mean walking intensity with additional mutual adjustment for walking intensity in model A (upper panel) and daily walking volume in model B (lower panel) **eTable 4.** Risk of chronic low back pain associated with quarters of daily walking volume and quarters of mean walking intensity with mutual adjustment for walking intensity and walking volume **eTable 5.** Risk of chronic low back pain associated with quarters of daily walking volume and quarters of mean walking intensity excluding participants reporting a history of chronic diseases at baseline **eTable 6.** Risk of chronic low back pain associated with quarters of daily walking volume and quarters of mean walking intensity excluding participants reporting “not so good” or “poor” health status at baseline **eTable 7.** Risk of chronic low back pain associated with quarters of daily walking volume and quarters of mean walking intensity excluding participants reporting any chronic pain at baseline **eTable 8.** Risk of chronic low back pain associated with quarters of daily walking volume and quarters of mean walking intensity excluding participants reporting moderate level of pain or stronger at baseline **eTable 9.** Risk of chronic low back pain associated with quarters of daily walking volume and quarters of mean walking intensity with additional adjustment for body mass index **eTable 10.** Risk of chronic low back pain associated with quarters of daily walking volume and quarters of mean walking intensity excluding participants with <4 valid days of accelerometry at baseline **eTable 11.** Risk of chronic low back pain associated with quarters of daily walking volume and quarters of mean walking intensity with additional adjustment for other physical activities **eTable 12.** Risk of chronic low back pain combined with limitations in spare time activities associated with quarters of daily walking volume and quarters of mean walking intensity **eTable 13.** Baseline characteristics of the study population stratified by quarters of daily walking volume including exercise frequency and physical work demands **eTable 14.** Baseline characteristics at HUNT4 among included and non-included participants **eTable 15.** Baseline characteristics at HUNT4 among participants with and without accelerometer data **eTable 16.** Baseline characteristics at HUNT4 among participants with and without chronic pain data

Supplement 2.
Data Sharing Statement
