# Supplementary material for: Volume and Intensity of Walking and Risk of Chronic Low Back Pain
Source: JAMA Netw Open. 2025 Jun 13;8(6):e2515592. doi: 10.1001/jamanetworkopen.2025.15592 (PMC12166487; doi:10.1001/jamanetworkopen.2025.15592)
Supplement: Supplement 1. — eTable 1. Selected conversion values for walking intensity eReference eFigure 1. Directed acyclic graph for covariate selection eTable 2. Risk of chronic low back pain associated with quarters of daily walking volume stratified by age eTable 3. Risk of chronic low back pain associated with quarters of mean walking intensity stratified by quarters of daily walking volume eFigure 2. Dose-response association of volume of daily walking and mean walking intensity with additional mutual adjustment for walking intensity in model A (upper panel) and daily walking volume in model B (lower panel) eTable 4. Risk of chronic low back pain associated with quarters of daily walking volume and quarters of mean walking intensity with mutual adjustment for walking intensity and walking volume eTable 5. Risk of chronic low back pain associated with quarters of daily walking volume and quarters of mean walking intensity excluding participants reporting a history of chronic diseases at baseline eTable 6. Risk of chronic low back pain associated with quarters of daily walking volume and quarters of mean walking intensity excluding participants reporting “not so good” or “poor” health status at baseline eTable 7. Risk of chronic low back pain associated with quarters of daily walking volume and quarters of mean walking intensity excluding participants reporting any chronic pain at baseline eTable 8. Risk of chronic low back pain associated with quarters of daily walking volume and quarters of mean walking intensity excluding participants reporting moderate level of pain or stronger at baseline eTable 9. Risk of chronic low back pain associated with quarters of daily walking volume and quarters of mean walking intensity with additional adjustment for body mass index eTable 10. Risk of chronic low back pain associated with quarters of daily walking volume and quarters of mean walking intensity excluding participants with <4 valid days of accelerometry at baseline eTable 11. Risk of chronic [file jamanetwopen-e2515592-s001.pdf]

Supplemental Online Content

Haddadj R, Nordstoga AL, Nilsen TIL, et al. Volume and intensity of walking and risk of chronic low back pain. *JAMA Netw Open*. 2025;8(6):e2515592. doi:10.1001/jamanetworkopen.2025.15592

eTable 1. Selected conversion values for walking intensity

eReference

eFigure 1. Directed acyclic graph for covariate selection

eTable 2. Risk of chronic low back pain associated with quarters of daily walking volume stratified by age

eTable 3. Risk of chronic low back pain associated with quarters of mean walking intensity stratified by quarters of daily walking volume

eFigure 2. Dose-response association of volume of daily walking and mean walking intensity with additional mutual adjustment for walking intensity in model A (upper panel) and daily walking volume in model B (lower panel)

eTable 4. Risk of chronic low back pain associated with quarters of daily walking volume and quarters of mean walking intensity with mutual adjustment for walking intensity and walking volume

eTable 5. Risk of chronic low back pain associated with quarters of daily walking volume and quarters of mean walking intensity excluding participants reporting a history of chronic diseases at baseline

eTable 6. Risk of chronic low back pain associated with quarters of daily walking volume and quarters of mean walking intensity excluding participants reporting “not so good” or “poor” health status at baseline

eTable 7. Risk of chronic low back pain associated with quarters of daily walking volume and quarters of mean walking intensity excluding participants reporting any chronic pain at baseline

eTable 8. Risk of chronic low back pain associated with quarters of daily walking volume and quarters of mean walking intensity excluding participants reporting moderate level of pain or stronger at baseline

eTable 9. Risk of chronic low back pain associated with quarters of daily walking volume and quarters of mean walking intensity with additional adjustment for body mass index

eTable 10. Risk of chronic low back pain associated with quarters of daily walking volume and quarters of mean walking intensity excluding participants with <4 valid days of accelerometry at baseline

eTable 11. Risk of chronic low back pain associated with quarters of daily walking volume and quarters of mean walking intensity with additional adjustment for other physical activities

© 2025 Haddadj R et al. *JAMA Network Open*.

eTable 12. Risk of chronic low back pain combined with limitations in spare time activities associated with quarters of daily walking volume and quarters of mean walking intensity

eTable 13. Baseline characteristics of the study population stratified by quarters of daily walking volume including exercise frequency and physical work demands

eTable 14. Baseline characteristics at HUNT4 among included and non-included participants

eTable 15. Baseline characteristics at HUNT4 among participants with and without accelerometer data

eTable 16. Baseline characteristics at HUNT4 among participants with and without chronic pain data

This supplemental material has been provided by the authors to give readers additional information about their work.

© 2025 Haddadj R et al. *JAMA Network Open*.

eTable 1. Selected conversion values for walking intensity

Physical Activity Compendium (PAC) activity description[1]	PAC code	PAC estimated MET value	Current study activity description
Walking, 2.0 to 2.4 mph, level, slow pace, firm surface	17152	2.8	Slow walking ≤4 km/h
Walking, 2.8 to 3.4 mph, level, moderate pace, firm surface	17190	3.8	Moderate walking 4.1-5.4 km/h
Walking, 3.5 to 3.9 mph, level, brisk, firm surface, walking for exercise	17200	4.8	Brisk walking 5.5-6.4 km/h

Abbreviation: MET, metabolic equivalent of task

eReference

1. Herrmann SD, Willis EA, Ainsworth BE, et al. 2024 Adult Compendium of Physical Activities: A third update of the energy costs of human activities. *J Sport Health Sci*. 2024;13(1):6-12. doi:10.1016/j.jshs.2023.10.010

© 2025 Haddadj R et al. *JAMA Network Open*.

eFigure 1. Directed acyclic graph for covariate selection

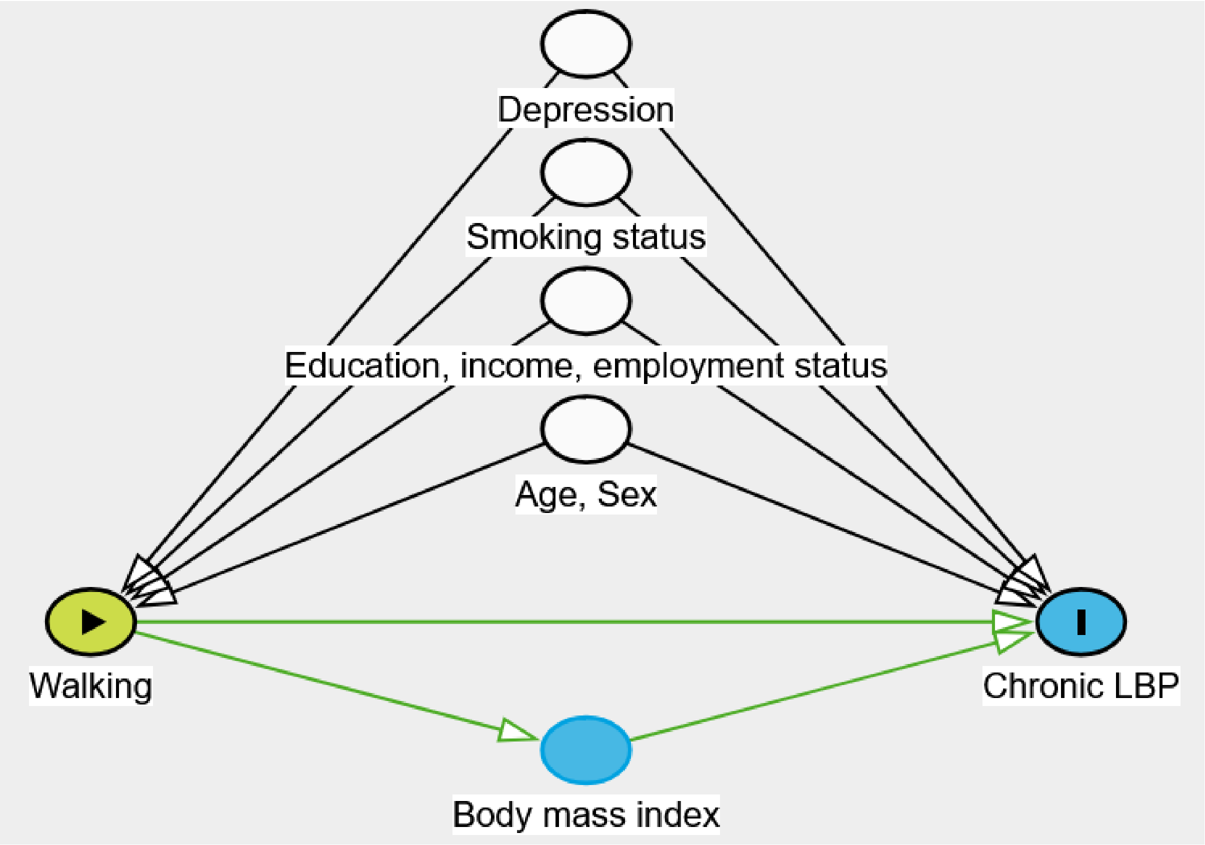

eTable 2. Risk of chronic low back pain associated with quarters of daily walking volume stratified by age

	No. of participants/ No. of cases	Age-adjusted, RR (95% CI)[a]	Multi-adjusted, RR (95% CI)[b]
Age <65 (n = 7 757)			
Quarter 1	1654/259	1.00 (ref)	1.00 (ref)
Quarter 2	1985/274	0.87 (0.75-1.02)	0.92 (0.79-1.08)
Quarter 3	2008/249	0.78 (0.66-0.92)	0.82 (0.70-0.97)
Quarter 4	2110/280	0.83 (0.71-0.97)	0.88 (0.75-1.02)
Age ≥65 (n = 3 437)			
Quarter 1	1146/260	1.00 (ref)	1.00 (ref)
Quarter 2	815/144	0.82 (0.68-0.98)	0.86 (0.71-1.03)
Quarter 3	788/115	0.69 (0.56-0.85)	0.73 (0.59-0.89)
Quarter 4	688/78	0.55 (0.43-0.70)	0.59 (0.46-0.75)

Abbreviations: CI, confidence interval; RR, risk ratio.
[a] Adjusted for age (continuous).
[b] Adjusted for age (continuous), sex (female, male), education (primary school, 1-2 y of academic/vocational school, 3 y of academic/vocational school, 3-4 y vocational school/apprentice, university <4 y, university >4 y), income (<25 000, 25 000-45 000, 45 100-75 000, 75 100-100 000, >100 000 USD/y), employment status (employed, non-employed), smoking status (never, former, current) and depression (no, yes).

© 2025 Haddadj R et al. *JAMA Network Open*.

eTable 3. Risk of chronic low back pain associated with quarters of mean walking intensity stratified by quarters of daily walking volume

	No. of participants/ No. of cases	Age-adjusted, RR (95% CI)[a]	Multi-adjusted, RR (95% CI)[b]
Q1, walking volume			
Q1, walking intensity	1235/282	1.00 (ref)	1.00 (ref)
Q2, walking intensity	688/128	0.84 (0.70-1.01)	0.85 (0.71-1.03)
Q3, walking intensity	521/91	0.79 (0.64-0.98)	0.80 (0.64-1.00)
Q4, walking intensity	355/43	0.55 (0.41-0.74)	0.59 (0.43-0.79)
Q2, walking volume			
Q1, walking intensity	667/110	1.00 (ref)	1.00 (ref)
Q2, walking intensity	767/112	0.89 (0.70-1.13)	0.92 (0.73-1.17)
Q3, walking intensity	734/92	0.76 (0.59-0.98)	0.81 (0.63-1.05)
Q4, walking intensity	630/81	0.78 (0.60-1.01)	0.83 (0.64-1.09)
Q3, walking volume			
Q1, walking intensity	536/73	1.00 (ref)	1.00 (ref)
Q2, walking intensity	702/96	1.01 (0.76-1.34)	1.02 (0.77-1.35)
Q3, walking intensity	740/88	0.87 (0.65-1.16)	0.86 (0.64-1.14)
Q4, walking intensity	821/108	0.96 (0.73-1.27)	0.95 (0.72-1.26)
Q4, walking volume			
Q1, walking intensity	362/54	1.00 (ref)	1.00 (ref)
Q2, walking intensity	643/82	0.84 (0.61-1.15)	0.88 (0.64-1.21)
Q3, walking intensity	801/93	0.76 (0.56-1.04)	0.77 (0.56-1.05)
Q4, walking intensity	992/126	0.83 (0.61-1.11)	0.83 (0.61-1.11)

Abbreviations: CI, confidence interval; RR, risk ratio; Q, Quarter.

[a] Adjusted for age (continuous).

[b] Adjusted for age (continuous), sex (female, male), education (primary school, 1-2 y of academic/vocational school, 3 y of academic/vocational school, 3-4 y vocational school/apprentice, university <4 y, university >4 y), income (<25 000, 25 000-45 000, 45 100-75 000, 75 100-100 000, >100 000 USD/y), employment status (employed, non-employed), smoking status (never, former, current) and depression (no, yes).

© 2025 Haddadj R et al. *JAMA Network Open*.

eFigure 2. Dose-response association of volume of daily walking and mean walking intensity with additional mutual adjustment for walking intensity in model A (upper panel) and daily walking volume in model B (lower panel)

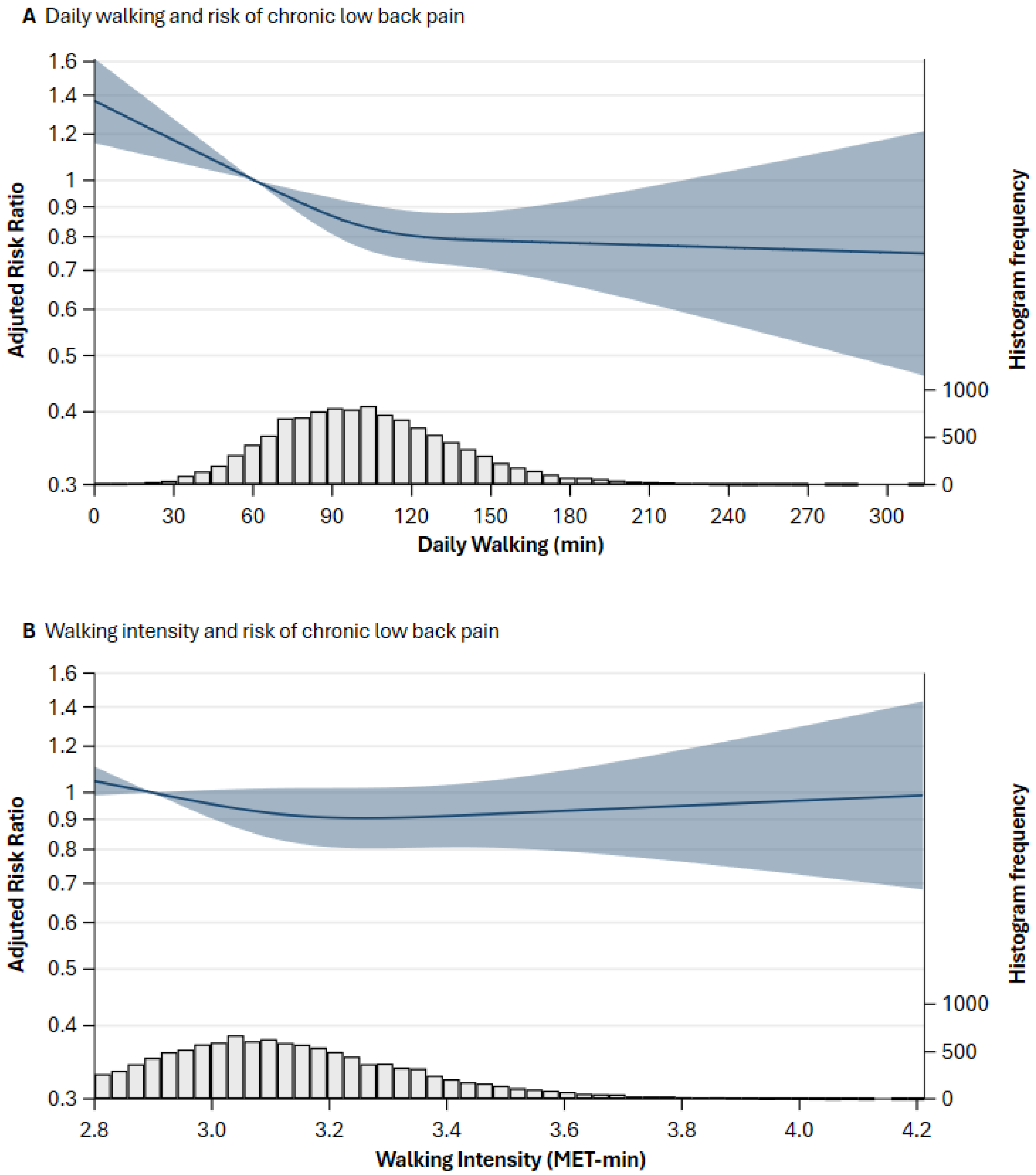

The left y-axis is a log scale with the shaded area representing 95% confidence interval. Both model A (upper panel) and model B (lower panel) are adjusted for age, sex, education, income, employment status, smoking status and depression. Additionally, model A is adjusted for mean walking intensity and model B is adjusted for daily walking volume. Reference is set at the 10th percentile of the distribution in both model A and model B. Abbreviation: MET, Metabolic Equivalent of Task.

© 2025 Haddadj R et al. *JAMA Network Open*.

eTable 4. Risk of chronic low back pain associated with quarters of daily walking volume and quarters of mean walking intensity with mutual adjustment for walking intensity and walking volume

	**No. of participants/ No. of cases**	**Age-adjusted, RR (95% CI)[a]**	**Multi-adjusted, RR (95% CI)[b]**
Daily walking volume			
Quarter 1	2800/519	1.00 (ref)	1.00 (ref)
Quarter 2	2800/418	0.83 (0.74-0.93)	0.88 (0.78-0.99)
Quarter 3	2796/364	0.72 (0.64-0.82)	0.78 (0.69-0.88)
Quarter 4	2798/358	0.71 (0.63-0.81)	0.78 (0.69-0.89)
Mean walking intensity			
Quarter 1	2799/544	1.00 (ref)	1.00 (ref)
Quarter 2	2798/395	0.77 (0.68-0.87)	0.88 (0.78-0.99)
Quarter 3	2799/365	0.72 (0.63-0.82)	0.86 (0.76-0.98)
Quarter 4	2798/355	0.70 (0.62-0.80)	0.88 (0.77-1.00)

Abbreviations: CI, confidence interval; RR, risk ratio.

[a] Adjusted for age (continuous).

[b] Adjusted for age (continuous), sex (female, male), education (primary school, 1-2 y of academic/vocational school, 3 y of academic/vocational school, 3-4 y vocational school/apprentice, university <4 y, university >4 y), income (<25 000, 25 000-45 000, 45 100-75 000, 75 100-100 000, >100 000 USD/y), employment status (employed, non-employed), smoking status (never, former, current) and depression (no, yes). Daily walking volume model is further adjusted for mean walking intensity (continuous). Mean walking intensity model is further adjusted for daily walking volume (continuous).

eTable 5. Risk of chronic low back pain associated with quarters of daily walking volume and quarters of mean walking intensity excluding participants reporting a history of chronic diseases* at baseline

	**No. of participants/ No. of cases**	**Age-adjusted, RR (95% CI)[a]**	**Multi-adjusted, RR (95% CI)[b]**
Daily walking volume			
Quarter 1	2160/347	1.00 (ref)	1.00 (ref)
Quarter 2	2157/295	0.86 (0.74-0.99)	0.89 (0.77-1.02)
Quarter 3	2159/272	0.79 (0.68-0.91)	0.82 (0.71-0.95)
Quarter 4	2158/271	0.78 (0.68-0.91)	0.82 (0.70-0.95)
Mean walking intensity			
Quarter 1	2159/371	1.00 (ref)	1.00 (ref)
Quarter 2	2158/271	0.76 (0.66-0.88)	0.83 (0.72-0.96)
Quarter 3	2159/288	0.81 (0.70-0.94)	0.91 (0.79-1.06)
Quarter 4	2158/255	0.72 (0.62-0.83)	0.83 (0.71-0.96)

*Variable composed of self-reported history diabetes or cancer or cardiovascular diseases
Abbreviations: CI, confidence interval; RR, risk ratio.
[a] Adjusted for age (continuous).
[b] Adjusted for age (continuous), sex (female, male), education (primary school, 1-2 y of academic/vocational school, 3 y of academic/vocational school, 3-4 y vocational school/apprentice, university <4 y, university >4 y), income (<25 000, 25 000-45 000, 45 100-75 000, 75 100-100 000, >100 000 USD/y), employment status (employed, non-employed), smoking status (never, former, current) and depression (no, yes).

© 2025 Haddadj R et al. *JAMA Network Open*.

eTable 6. Risk of chronic low back pain associated with quarters of daily walking volume and quarters of mean walking intensity excluding participants reporting "not so good" or "poor" health status at baseline

	**No. of participants/ No. of cases**	**Age-adjusted, RR (95% CI)[a]**	**Multi-adjusted, RR (95% CI)[b]**
Daily walking volume			
Quarter 1	2452/361	1.00 (ref)	1.00 (ref)
Quarter 2	2446/320	0.91 (0.79-1.05)	0.93 (0.81-1.07)
Quarter 3	2449/280	0.80 (0.69-0.92)	0.82 (0.71-0.95)
Quarter 4	2449/287	0.81 (0.71-0.94)	0.84 (0.73-0.97)
Mean walking intensity			
Quarter 1	2449/384	1.00 (ref)	1.00 (ref)
Quarter 2	2449/295	0.82 (0.71-0.94)	0.87 (0.75-0.99)
Quarter 3	2449/293	0.82 (0.71-0.95)	0.90 (0.78-1.04)
Quarter 4	2449/276	0.77 (0.67-0.89)	0.86 (0.74-0.99)

Abbreviations: CI, confidence interval; RR, risk ratio.
[a] Adjusted for age (continuous).
[b] Adjusted for age (continuous), sex (female, male), education (primary school, 1-2 y of academic/vocational school, 3 y of academic/vocational school, 3-4 y vocational school/apprentice, university <4 y, university >4 y), income (<25 000, 25 000-45 000, 45 100-75 000, 75 100-100 000, >100 000 USD/y), employment status (employed, non-employed), smoking status (never, former, current) and depression (no, yes).

© 2025 Haddadj R et al. *JAMA Network Open*.

eTable 7. Risk of chronic low back pain associated with quarters of daily walking volume and quarters of mean walking intensity excluding participants reporting any chronic pain at baseline

	**No. of participants/ No. of cases**	**Age-adjusted, RR (95% CI)[a]**	**Multi-adjusted, RR (95% CI)[b]**
Daily walking volume			
Quarter 1	1748/215	1.00 (ref)	1.00 (ref)
Quarter 2	1749/192	0.91 (0.76-1.10)	0.96 (0.80-1.15)
Quarter 3	1746/143	0.68 (0.56-0.83)	0.72 (0.59-0.89)
Quarter 4	1746/159	0.76 (0.62-0.92)	0.81 (0.66-0.98)
Mean walking intensity			
Quarter 1	1748/221	1.00 (ref)	1.00 (ref)
Quarter 2	1747/153	0.74 (0.60-0.90)	0.81 (0.66-0.99)
Quarter 3	1747/175	0.85 (0.70-1.03)	0.95 (0.79-1.16)
Quarter 4	1747/160	0.77 (0.64-0.94)	0.89 (0.73-1.08)

Abbreviations: CI, confidence interval; RR, risk ratio.
[a] Adjusted for age (continuous).
[b] Adjusted for age (continuous), sex (female, male), education (primary school, 1-2 y of academic/vocational school, 3 y of academic/vocational school, 3-4 y vocational school/apprentice, university <4 y, university >4 y), income (<25 000, 25 000-45 000, 45 100-75 000, 75 100-100 000, >100 000 USD/y), employment status (employed, non-employed), smoking status (never, former, current) and depression (no, yes).

© 2025 Haddadj R et al. *JAMA Network Open*.

eTable 8. Risk of chronic low back pain associated with quarters of daily walking volume and quarters of mean walking intensity excluding participants reporting moderate level of pain or stronger at baseline

	**No. of participants/ No. of cases**	**Age-adjusted, RR (95% CI)[a]**	**Multi-adjusted, RR (95% CI)[b]**
Daily walking volume			
Quarter 1	2038/285	1.00 (ref)	1.00 (ref)
Quarter 2	2040/238	0.86 (0.73-1.01)	0.89 (0.75-1.04)
Quarter 3	2036/187	0.68 (0.57-0.81)	0.71 (0.60-0.85)
Quarter 4	2037/206	0.74 (0.63-0.88)	0.78 (0.66-0.92)
Mean walking intensity			
Quarter 1	2038/280	1.00 (ref)	1.00 (ref)
Quarter 2	2038/214	0.82 (0.69-0.97)	0.88 (0.74-1.04)
Quarter 3	2038/214	0.83 (0.70-0.99)	0.91 (0.77-1.09)
Quarter 4	2037/208	0.80 (0.68-0.96)	0.90 (0.76-1.08)

Abbreviations: CI, confidence interval; RR, risk ratio.

[a] Adjusted for age (continuous).

[b] Adjusted for age (continuous), sex (female, male), education (primary school, 1-2 y of academic/vocational school, 3 y of academic/vocational school, 3-4 y vocational school/apprentice, university <4 y, university >4 y), income (<25 000, 25 000-45 000, 45 100-75 000, 75 100-100 000, >100 000 USD/y), employment status (employed, non-employed), smoking status (never, former, current) and depression (no, yes)

© 2025 Haddadj R et al. *JAMA Network Open*.

eTable 9. Risk of chronic low back pain associated with quarters of daily walking volume and quarters of mean walking intensity with additional adjustment for body mass index

	**No. of participants/ No. of cases**	**Age-adjusted, RR (95% CI)[a]**	**Multi-adjusted, RR (95% CI)[b]**
Daily walking volume			
Quarter 1	2794/519	1.00 (ref)	1.00 (ref)
Quarter 2	2790/417	0.83 (0.74-0.93)	0.90 (0.80-1.01)
Quarter 3	2792/364	0.72 (0.64-0.82)	0.81 (0.71-0.91)
Quarter 4	2792/357	0.71 (0.63-0.80)	0.82 (0.72-0.93)
Mean walking intensity			
Quarter 1	2792/545	1.00 (ref)	1.00 (ref)
Quarter 2	2792/394	0.77 (0.68-0.87)	0.87 (0.77-0.98)
Quarter 3	2792/364	0.72 (0.63-0.81)	0.86 (0.75-0.97)
Quarter 4	2792/354	0.70 (0.62-0.79)	0.87 (0.76-0.99)

Abbreviations: CI, confidence interval; RR, risk ratio.

[a] Adjusted for age (continuous).

[b] Adjusted for age (continuous), sex (female, male), education (primary school, 1-2 y of academic/vocational school, 3 y of academic/vocational school, 3-4 y vocational school/apprentice, university <4 y, university >4 y), income (<25 000, 25 000-45 000, 45 100-75 000, 75 100-100 000, >100 000 USD/y), employment status (employed, non-employed), smoking status (never, former, current) and depression (no, yes) and body mass index (continuous).

© 2025 Haddadj R et al. *JAMA Network Open*.

eTable 10. Risk of chronic low back pain associated with quarters of daily walking volume and quarters of mean walking intensity excluding participants with <4 valid days of accelerometry at baseline

	**No. of participants/ No. of cases**	**Age-adjusted, RR (95% CI)[a]**	**Multi-adjusted, RR (95% CI)[b]**
Daily walking volume			
Quarter 1	2649/487	1.00 (ref)	1.00 (ref)
Quarter 2	2650/385	0.82 (0.73-0.93)	0.86 (0.76-0.97)
Quarter 3	2648/331	0.71 (0.62-0.80)	0.75 (0.65-0.85)
Quarter 4	2647/335	0.72 (0.63-0.81)	0.76 (0.67-0.87)
Mean walking intensity			
Quarter 1	2649/487	1.00 (ref)	1.00 (ref)
Quarter 2	2648/385	0.75 (0.66-0.85)	0.82 (0.72-0.93)
Quarter 3	2649/331	0.72 (0.63-0.82)	0.81 (0.71-0.92)
Quarter 4	2648/335	0.70 (0.61-0.80)	0.81 (0.71-0.93)

Abbreviations: CI, confidence interval; RR, risk ratio.
[a] Adjusted for age (continuous).
[b] Adjusted for age (continuous), sex (female, male), education (primary school, 1-2 y of academic/vocational school, 3 y of academic/vocational school, 3-4 y vocational school/apprentice, university <4 y, university >4 y), income (<25 000, 25 000-45 000, 45 100-75 000, 75 100-100 000, >100 000 USD/y), employment status (employed, non-employed), smoking status (never, former, current) and depression (no, yes).

© 2025 Haddadj R et al. *JAMA Network Open*.

eTable 11. Risk of chronic low back pain associated with quarters of daily walking volume and quarters of mean walking intensity with additional adjustment for other physical activities

	No. of participants/ No. of cases	Age-adjusted, RR (95% CI)[a]	Multi-adjusted, RR (95% CI)[b]
Daily walking volume			
Quarter 1	2794/519	1.00 (ref)	1.00 (ref)
Quarter 2	2790/417	0.83 (0.74-0.93)	0.87 (0.78-0.98)
Quarter 3	2792/364	0.72 (0.64-0.82)	0.77 (0.68-0.87)
Quarter 4	2792/357	0.71 (0.63-0.81)	0.77 (0.68-0.87)
Mean walking intensity			
Quarter 1	2792/545	1.00 (ref)	1.00 (ref)
Quarter 2	2792/394	0.77 (0.68-0.87)	0.85 (0.75-0.96)
Quarter 3	2792/364	0.72 (0.63-0.82)	0.82 (0.72-0.93)
Quarter 4	2792/354	0.70 (0.62-0.80)	0.82 (0.72-0.94)

Abbreviations: CI, confidence interval; RR, risk ratio.

[a] Adjusted for age (continuous).

[b] Adjusted for age (continuous), sex (female, male), education (primary school, 1-2 y of academic/vocational school, 3 y of academic/vocational school, 3-4 y vocational school/apprentice, university <4 y, university >4 y), income (<25 000, 25 000-45 000, 45 100-75 000, 75 100-100 000, >100 000 USD/y), employment status (employed, non-employed), smoking status (never, former, current) and depression (no, yes) and other physical activities (continuous).

© 2025 Haddadj R et al. *JAMA Network Open*.

eTable 12. Risk of chronic low back pain combined with limitations in spare time activities associated with quarters of daily walking volume and quarters of mean walking intensity

	**No. of participants/ No. of cases**	**Age-adjusted, RR (95% CI)[a]**	**Multi-adjusted, RR (95% CI)[b]**
Daily walking volume			
Quarter 1	2800/250	1.00 (ref)	1.00 (ref)
Quarter 2	2800/193	0.78 (0.65-0.93)	0.83 (0.69-0.99)
Quarter 3	2796/167	0.67 (0.56-0.81)	0.73 (0.60-0.88)
Quarter 4	2798/143	0.58 (0.47-0.70)	0.64 (0.52-0.78)
Mean walking intensity			
Quarter 1	2799/269	1.00 (ref)	1.00 (ref)
Quarter 2	2798/180	0.66 (0.55-0.80)	0.73 (0.61-0.88)
Quarter 3	2799/155	0.57 (0.47-0.69)	0.66 (0.54-0.80)
Quarter 4	2798/149	0.55 (0.45-0.66)	0.64 (0.53-0.79)

Abbreviations: CI, confidence interval; RR, risk ratio.
[a] Adjusted for age (continuous).
[b] Adjusted for age (continuous), sex (female, male), education (primary school, 1-2 y of academic/vocational school, 3 y of academic/vocational school, 3-4 y vocational school/apprentice, university <4 y, university >4 y), income (<25 000, 25 000-45 000, 45 100-75 000, 75 100-100 000, >100 000 USD/y), employment status (employed, non-employed), smoking status (never, former, current) and depression (no, yes).

© 2025 Haddadj R et al. *JAMA Network Open*.

eTable 13. Baseline characteristics of the study population stratified by quarters of daily walking volume including exercise frequency and physical work demands

	**Daily walking volume, No. (%)**				
**Characteristic**	**Total**	**Q1 (<78 min/day)**	**Q2 (78-100 min/day)**	**Q3 (101-124 min/day)**	**Q4 (≥125 min/day)**
Participants, No.	2800	2800	2796	2798	11194
Age, mean (SD), y	57.4 (17.3)	54.6 (15.3)	54.7 (14.3)	54.6 (13.1)	55.3 (15.1)
Female	1570 (56.1)	1659 (59.2)	1703 (60.9)	1632 (58.3)	6564 (58.6)
Valid days of accelerometry, mean (SD)	5.6 (1.1)	5.7 (1.0)	5.7 (0.9)	5.7 (1.1)	5.7 (1.0)
Walking intensity, mean (SD), METs-min	3.1 (0.2)	3.1 (0.2)	3.2 (0.2)	3.2 (0.2)	3.1 (0.2)
Exercise frequency[a]					
Never	89 (3.2)	56 (2.0)	37 (1.3)	38 (1.4)	220 (2.0)
<1 time a week	451 (16.3)	300 (10.8)	203 (7.3)	180 (6.5)	1134 (10.2)
Once a week	575 (20.7)	462 (16.6)	408 (14.8)	356 (12.8)	1801 (16.2)
2-3 times a week	1275 (46.0)	1364 (49.1)	1371 (49.6)	1185 (42.7)	5195 (46.9)
Nearly every day	383 (13.8)	597 (21.5)	743 (26.9)	1015 (36.6)	2738 (24.7)
Physical work demands[b]					
Non-employed	1329 (47.6)	867 (31.1)	807 (29.0)	679 (24.4)	3682 (33.0)
Mostly sedentary	833 (29.9)	868 (31.1)	718 (25.8)	511 (18.3)	2930 (26.3)
Lot of walking	341 (12.2)	584 (20.9)	684 (24.6)	768 (27.6)	2377 (21.3)
Lot of walking and lifting	252 (9.0)	418 (15.0)	512 (18.4)	674 (24.2)	1856 (16.6)
Heavy manual labour	35 (1.3)	53 (1.9)	63 (2.3)	155 (5.6)	306 (2.7)

[a] Self-report item asking: "How often do you exercise?"
[b] Response to the question "If you have paid or unpaid work: How would you describe your work?"

© 2025 Haddadj R et al. *JAMA Network Open*.

eTable 14. Baseline characteristics at HUNT4 among included and non-included participants

	**HUNT4 participants**	**Included participants**	**Non-included participants**
	**No. (%)**		
Participants, No.	56040	11194	44846
Age, mean (SD), y	54.6 (17.6)	55.3 (15.1)	54.5 (18.1)
Female	30575 (54.6)	6564 (58.6)	24011 (53.5)
Education			
Primary school	6499 (11.7)	719 (6.4)	5780 (13.0)
Secondary school	27405 (49.3)	5029 (44.9)	22376 (50.5)
University	21628 (38.9)	5446 (48.7)	16182 (36.5)
Yearly household income			
<25 000 USD	5620 (10.4)	475 (4.2)	5145 (12.0)
25 000-45 000 USD	11736 (21.7)	1969 (17.6)	9767 (22.7)
45 100-75 000 USD	15750 (29.1)	3418 (30.5)	12332 (28.7)
75 100-100 000 USD	11521 (21.3)	2881 (25.7)	8640 (20.1)
>100 000 USD	9563 (17.6)	2451 (21.9)	7112 (16.5)
Employment status			
Employed	33124 (61.9)	7512 (67.1)	25612 (60.5)
Non-employed	20418 (38.1)	3682 (32.9)	16736 (39.5)
Smoking status			
Never smoker	24569 (44.2)	5284 (47.2)	19285 (43.4)
Former smoker	25509 (45.8)	5165 (46.1)	20344 (45.8)
Current smoker	5560 (10.0)	745 (6.7)	4815 (10.8)
Depression[a]			
No	35394 (85.8)	10090 (90.1)	25304 (84.2)
Yes	5847 (14.2)	1104 (9.9)	4743 (15.8)

Abbreviations: USD, United States dollar.
[a] Depression was assessed using the depression subscale of the Hospital Anxiety and Depression Scale.

© 2025 Haddadj R et al. *JAMA Network Open*.

eTable 15. Baseline characteristics at HUNT4 among participants with and without accelerometer data

	**HUNT4 participants**	**No (valid) accelerometer data**	**Valid accelerometer data**
	**No. (%)**		
Participants, No.	56040	28712	27328
Age, mean (SD), y	54.6 (17.6)	55.3 (18.2)	54.0 (16.9)
Female,	30575 (54.6)	15042 (52.4)	15533 (56.8)
Education			
Primary school	6499 (11.7)	3968 (14.0)	2531 (9.3)
Secondary school	27405 (49.3)	14463 (51.1)	12942 (47.6)
University	21628 (38.9)	9900 (34.9)	11728 (43.1)
Yearly household income			
<25 000 USD	5620 (10.4)	3423 (12.4)	2197 (8.2)
25 000-45 000 USD	11736 (21.7)	6223 (22.6)	5513 (20.7)
45 100-75 000 USD	15750 (29.1)	7851 (28.6)	7899 (29.6)
75 100-100 000 USD	11521 (21.3)	5523 (20.1)	5998 (22.5)
>100 000 USD	9563 (17.6)	4478 (16.3)	5085 (19.1)
Employment status			
Employed	33124 (61.9)	15747 (59.5)	17377 (64.2)
Non-employed	20418 (38.1)	10722 (40.5)	9696 (35.8)
Smoking status			
Never smoker	24569 (44.2)	12302 (43.3)	12267 (45.1)
Former smoker	25509 (45.8)	12937 (45.5)	12572 (46.2)
Current smoker	5560 (10.0)	3199 (11.2)	2361 (8.7)
Depression[a]			
No	35394 (85.8)	15326 (84.9)	20068 (86.6)
Yes	5847 (14.2)	2736 (15.1)	3111 (13.4)

Abbreviations: USD, United States dollar.

[a] Depression was assessed using the depression subscale of the Hospital Anxiety and Depression Scale.

© 2025 Haddadj R et al. *JAMA Network Open*.

eTable 16. Baseline characteristics at HUNT4 among participants with and without chronic pain data

	**HUNT4 participants**	**Missing information on chronic pain**	**Complete information on chronic pain**
	**No. (%)**		
Participants, No.	56040	14600	41440
Age, mean (SD), y	54.6 (17.6)	50.7 (18.9)	55.9 (16.9)
Female	30575 (54.6)	6964 (47.7)	23611 (57.0)
Education			
Primary school	6499 (11.7)	1776 (12.4)	4723 (11.5)
Secondary school	27405 (49.3)	7286 (50.9)	20119 (48.8)
University	21628 (38.9)	5255 (36.7)	16373 (39.7)
Yearly household income			
<25 000 USD	5620 (10.4)	1920 (13.9)	3700 (9.2)
25 000-45 000 USD	11736 (21.7)	2889 (20.9)	8847 (21.9)
45 100-75 000 USD	15750 (29.1)	3634 (26.2)	12116 (30.0)
75 100-100 000 USD	11521 (21.3)	2867 (20.7)	8654 (21.5)
>100 000 USD	9563 (17.6)	2536 (18.3)	7027 (17.4)
Employment status			
Employed	33124 (61.9)	8550 (67.4)	24574 (60.2)
Non-employed	20418 (38.1)	4144 (32.6)	16274 (39.8)
Smoking status			
Never smoker	24569 (44.2)	6461 (44.9)	18108 (43.9)
Former smoker	25509 (45.8)	6270 (43.6)	19239 (46.6)
Current smoker	5560 (10.0)	1656 (11.5)	3904 (9.5)
Depression[a]			
No	35394 (85.8)	748 (84.5)	34646 (85.9)
Yes	5847 (14.2)	137 (15.5)	5710 (14.1)

Abbreviations: USD, United States dollar.

[a] Depression was assessed using the depression subscale of the Hospital Anxiety and Depression Scale.

© 2025 Haddadj R et al. *JAMA Network Open*.
