# Supplementary material for: Volume and Intensity of Walking and Risk of Chronic Low Back Pain
Source: JAMA Netw Open. 2025 Jun 13;8(6):e2515592. doi: 10.1001/jamanetworkopen.2025.15592 (PMC12166487; doi:10.1001/jamanetworkopen.2025.15592)
Supplement: Supplement 2. — Data Sharing Statement [file jamanetwopen-e2515592-s002.pdf]

Data Sharing Statement

Haddadj. Volume and Intensity of Walking and Risk of Chronic Low Back Pain. *JAMA Netw Open*. Published June 13, 2025. doi:10.1001/jamanetworkopen.2025.15592

Data
Data available: No

Additional Information
Explanation for why data not available: The data that support the findings of this study are available from HUNT Research Centre (https://www.ntnu.edu/hunt) but restrictions apply to the availability of these data, which were used under license for the current study, and so are not publicly available. Data are however available from the co-author "Paul Jarle Mork" (Email ID: paul.mork@ntnu.no) upon reasonable request and with permission from HUNT Research Centre (https://www.ntnu.edu/hunt).
